# Never-homozygous genetic variants in healthy populations are potential recessive disease candidates

**DOI:** 10.1038/s41525-022-00322-z

**Published:** 2022-09-08

**Authors:** Torsten Schmenger, Gaurav D. Diwan, Gurdeep Singh, Gordana Apic, Robert B. Russell

**Affiliations:** 1 BioQuant, Heidelberg University, Heidelberg, Germany; 2 Heidelberg University Biochemistry Center (BZH), Heidelberg, Germany

**Keywords:** Data mining, Neurodegenerative diseases, Disease genetics

## Abstract

The rapid pace with which genetic variants are now being determined means there is a pressing need to understand how they affect biological systems. Variants from healthy individuals have previously been used to study blood groups or HLA diversity and to identify genes that can apparently be nonfunctional in healthy people. These studies and others have observed a lower than expected frequency of homozygous individuals for potentially deleterious alleles, which would suggest that several of these alleles can lead to recessive disorders. Here we exploited this principle to hunt for potential disease variants in genomes from healthy people. We identified at least 108 exclusively heterozygous variants with evidence for an impact on biological function. We discuss several examples of candidate variants/genes including CCDC8, PANK3, RHD and NLRP12. Overall, the results suggest there are many, comparatively frequent, potentially lethal or disease-causing variants lurking in healthy human populations.

## Introduction

**Fig. 1:**
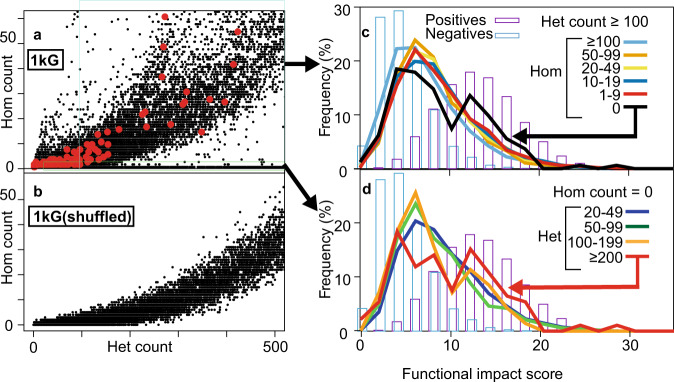
Exclusively heterozygous variants in 1 kG. **a** Plots of homozygous vs heterozygous counts for the 1 kG dataset. The preponderance of values on the X axis (i.e. zero homozygous counts) are indicated. **b** As in a) but with shuffled 1 kG data. **c** How the distribution of functional impact scores changes as homozygous counts decrease. **d** How the distribution of functional impact scores changes for sites where homozygous counts are zero with increasing heterozygous counts.

An analysis of counts of homozygous versus heterozygous among the 201k single nucleotide, missense variants from the 1000 Genome Project (1 kG)^1^ shows a general increasing proportion of homozygotes (Fig. 1a) that also agrees with simulated datasets (Fig. 1b, see Methods). Mendelian inheritance suggests that the degree of homozygosity should be 8–29%, which in turn dictates that positions with variants in ≥41 genomes (in 1 kG) would be unlikely to have zero homozygous (hom) counts. Indeed, in simulations we see very few exclusively homozygous positions above this value and none at all above 259. After removing positions where 1 kG and gnomAD^1,2^ datasets wildly disagreed in terms of minor allele frequencies, we had 167k variants of which 1943 have zero hom counts and total variant count ≥41 in the 1 kG dataset, of which 38 also satisfy the same requirement within gnomAD. We reasoned that errors were more likely in the more heterogeneous gnomAD dataset, so we tolerated a hom count ≤ 5 to derive a set of 353 variants. Lowering this threshold gives values of 223, 156, 112, 75 and 50 (for values 4 through 0). This threshold is, of course, somewhat arbitrary as we do not have any benchmark (or indeed knowledge) of the phenomenon we are investigating. More conservative sets can be extracted from the dataset in Supplementary Table 1.

**Fig. 2:**
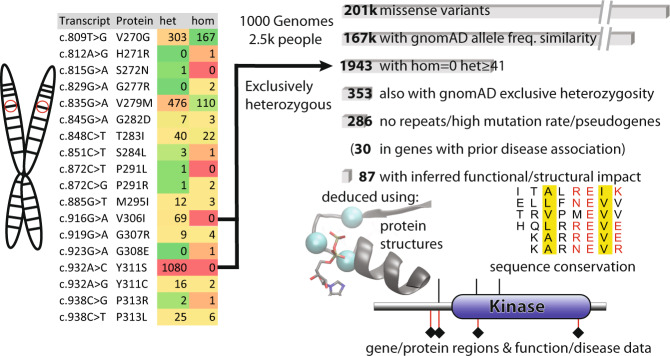
Filtering and data processing overview. Overview showing the processing and filtering of 201k missense variants based on exclusive heterozygosity.

We also excluded genes that are highly repeat-prone, unusually subject to mutations or that showed any hints of the existence of pseudogenes that might obscure the signal (specifically Filaggrin, Mucins, Olfactory receptors and Rootletin). This then gave a final set of 286 exclusively heterozygous variants that we considered further. A summary of how we defined and filtered the data to arrive at these variants is given in Fig. 2.

We studied these 286 exclusively heterozygous variants to test if the phenomenon might be explained by various gene features. They show no obvious pattern in terms of chromosomal location and there are no clear clusters around any loci. We believe this rules out a haplotype-based explanation for their occurrence. The genes also show no increased tendency (compared to others) in their tolerance of loss-of-function variants (as defined using pLI or pRec from gnomAD). We also found no significant difference between these genes, 3586 disease genes (those with at least one Mendelian variant defined in UniProt/OMIM) or all other genes when comparing variant profiles, defined by the number of individuals (1 kG) that contained homozygous, heterozygous or compound heterozygous variants at any position.

For comparison, we also considered other variant types than missense. The 2k frame-shift variants, in-frame insertions/deletions and stop-gains (Supplementary Figure 1a-c) have a number of exclusively heterozygous variants as might be expected as they are likely to alter/abate protein function^3^. Reassuringly however, we see significantly fewer exclusively heterozygous splice (Wilcoxon rank sum test, p = 2.69×10^−12^), intronic (*p* = 2.78×10^−12^), UTR (*p* = 9.1×10^−8^), synonymous (*p* < 2.2×10^−16^) or non-coding (*p* = 2.55×10^−11^, Supplementary Figure 1d-h) variants compared to missense. Overall, these observations support the notion that many of our observed exclusively heterozygous missense variants could be functional.

Interestingly 30 of these exclusively heterozygous missense variants lie in 28 known disease genes^4^, but are currently not known to be causative (see examples below). However, the majority of variants (256) are in genes not currently associated with disease.

We used several metrics to assess the structural and functional impact of the above variants (Fig. 2). These included conservation across sets of orthologs or human paralogs, structural measurements derived from Alphafold2 structures^5,6^, functional information from UniProt^7^, haplotype insufficiency, previous observations of gene-disease association and others (see Methods). The Bayesian combination of scores gave a performance on a benchmark set roughly in line with the best previously published predictors (Supplementary Figure 2b). We avoided including the existing methods^8–10^ into the integrated score as several of our candidate variants (i.e. common SNPs) are included in the negatives used to benchmark the respective methods.

The degree of heterozygous exclusivity appears to enrich for functional impact. For example, for all 1 kG variants with zero homozygous counts, the fraction of those having a predicted functional impact increases with the heterozygous count (Fig. 1d – red line). In addition, increasing the homozygous count above zero diminishes the fraction that have a predicted impact (Fig. 1c – black line). Both of these observations suggest that the degree of exclusive heterozygosity enriches for functionally disruptive variants thus implying a fraction of these positions are likely to disrupt or modify protein function. Both of these results are also seen when using previously published variant impact predictors (e.g. PMUT, Supplementary Figure 2d), though to a lesser degree. Of the 286 exclusively heterozygous variants, 87 (30.4%) have a functional impact score (≥11) with a false-discovery rate < 1% and a false positive rate < 5%, also suggesting a substantial enrichment of functionally relevant changes.

As might be expected, these 87 genes show little coherence in terms of function (no significant enrichment via DAVID^11^ or GetGo^12^), though certain broad groups are apparent in (albeit insignificant) gene-enrichment output, protein function (Uniprot) and the literature. Interestingly, these are conditions that might lead to symptoms in single tissues later in life (e.g. eyes or kidneys in the ciliopathies) or those that might only manifest under certain circumstances (autoimmunity or obesity). Below, we discuss six examples in more detail we found convincing after inspection of existing information on gene/protein function, associated diseases and/or other disease variants was available. The full list of 286 variants is given in Supplementary Table 1.

**Fig. 3:**
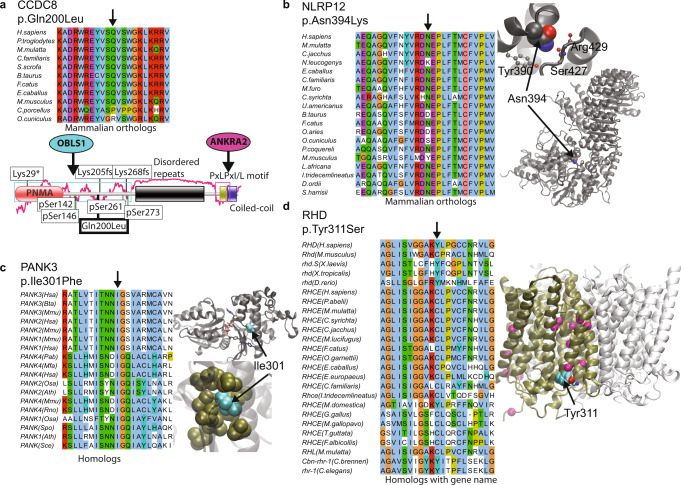
Examples of exclusively heterozygous variants. Examples of exclusively heterozygous variants showing hints of a possible structural/functional consequence. **a** Top: Jalview^78^ alignment of selected mammalian orthologs around Gln200 (Arrow) in CCDC8. Conserved residues are shown in ClustalX colours. Bottom: domain diagram superimposed on top of a IUPred plot of protein disorder^15^. Locations of phosphorylated serines and other mutations associated with disease are labelled in addition to p.Gln200Leu. **b** Left: as for a). Right: VMD^79^ representation of the Alphafold2^6^ NLRP12 model showing the location of Asn394. The zoomed view highlights (ball-and-stick representation) sidechains (Tyr390) or mainchain (Ser427 Arg429) atoms in contact with Asn394 (spheres). **c** Left: alignment as for a) but with PANK1-3 paralogs from Uniprot Sprot. Right: VMD representation of location of PANK3 Ile301 on the crystal structure (RCSB PDB:6pe6). The zoomed image shows how PANK3 (cyan spheres) packs tightly against hydrophobic sidechains (brown). **d** Left: alignment as for c). Right: VMD representation of a superimposition^80^ of the Alphafold2 structure of RHD superimposed with two copies of RHCG (using RCSB PDB:3hd6). The location of Tyr311 is shown (cyan/red spheres) as are the Ca atoms of residues harbouring weak D mutations (magenta).

Among variants potentially affecting ciliary processes is p.Gln200Leu in coiled-coil domain-containing protein 8 (CCDC8; Fig. 3a), which is exclusively heterozygous in 44 (1.8%) 1 kG participants as well as in 694 (0.26 %) gnomAD individuals (though with 6 homozygous instances). CCDC8, together with CUL7 and OBSL1 forms the 3 M complex involved in regulating microtubule dynamics and genome integrity^13^. Mutually exclusive, homozygous or compound heterozygous mutations in these three genes are causative of 3 M syndrome^13^, an autosomal recessive growth disorder with prenatal growth restriction and the failure of postnatal catch-up, resulting in short stature and skeletal abnormalities^14^ and a likely ciliopathy^12^. Gln200 is largely conserved in vertebrates and lies within a short ordered segment^15^. *CCDC8*-null mice showed defects in trophoblast motility known to result in complications during pregnancy such as placentation failures or even fetal death^16^ Other known CCDC8 3 M syndrome mutations are stop-gains or frameshifts, though there are 3 M missense mutations in CUL7^17^. It has been proposed that these CCDC8 3 M mutations disrupt the binding of ANKRA2, which has been shown to recognize a C-terminal motif in CCDC8 (Fig. 3a)^18^. Gln200 lies in a putative WW domain region that is phosphorylated and indeed high-throughput studies have identified putative phosphorylation events at Tyr197 and Ser202^19^. Nearby phosphorylations are thought to mediate interactions with other 3 M proteins^16^; indicating that p.Gln200Leu might disrupt folding or interactions involving this region of CCDC8.

Another candidate variant affecting ciliary function is p.Pro420Leu in the transcription factor GLIS2, which is exclusively heterozygous in 44 (1.7%) 1 kG participants and in 255 (0.1%) in gnomAD individuals (with 3 homozygous instances). There is no known or confidently predicted structure for this region of GLIS2, though Pro420 is largely conserved across homologs. GLIS2 has recently been reported to have important functions in cellular reprogramming^20,21^ and a fusion of CBFA2T3 to GLIS2 is frequently observed in early onset acute leukaemia, often associated with poor prognosis^22,23^. Additional members of this protein family are also causative of diseases, for example GLI1 is causative of postaxial Polydactyly^24^, ZIC1 in Craniosynostosis^25^ and ZIC2 in Holoprosencephaly^26^. Interestingly, variants in GLIS2 are causative of Nephronophthisis, an end-stage kidney disease in children and young adults^27,28^.

Among the variants affecting proteins in the immune system is p.Asn394Lys in NLRP12 (Fig. 3b), a protein expressed in dendritic cells and macrophages^29^. This variant is exclusively heterozygous in 72 (2.9%) 1 kG and 70 (0.03%) gnomAD individuals. Asn394 lies within the NACHT domain of NLRP12 and is highly conserved in homologs (Fig. 3b). NLRP12 acts as a negative regulator of various inflammatory processes^30^. Loss-of-function, through deletions or frame-shift variants are associated with Familial cold autoinflammatory syndrome 2^31^, a disease triggered by exposure to cold with typical inflammatory symptoms (i.e. fever, rashes, myalgia and headaches).

Another candidate variant is p.Ala270Thr in Beta-1-Adrenergic Receptor 1 (ADRB1) which is exclusively heterozygous in 44 (1.8%) 1 kG and 353 (0.15%) gnomAD individuals (although with 2 homozygous instances). At least one variant in this protein (p.Arg389Gly) is associated with congestive heart failure^32^. ADRB1 plays roles in stimulate brown adipose tissue, suggesting that loss-of-function variants could progress to obesity and insulin resistance^33^. Both variants are located on the intracellular portion of the receptor (Ala270 is in the third intracellular loop, Arg389 is at the C-terminus), suggesting roles in G-protein recognition and signalling^34^. Ala270 is mostly conserved in mammals, though certain species have other residues, including at least one that has Thr at this position (Bottlenose dolphins).

The missense variant p.Ile301Phe from Pantothenate kinase 3 (PANK3; Fig. 3c) is exclusively heterozygous in 1 kG with 528 (21 %) and in gnomAD with 1820 (0.7%) carriers. PANK3 is one of three kinases essential in coenzyme A biosynthesis^35^ and are largely preserved across all three kingdoms of life. Loss-of-function variants in *S. cerevisae* and *D. melanogaster* are not viable^36^. Ile301 lies in the fumble domain^37^ buried in the core of the protein just under the active site of the enzyme. This position is either Isoleucine or Valine in all homologs of PANK1-3, suggesting that even the seemingly conservative change to Phenylalanine would not be tolerated (Fig. 3c). Defects in coenzyme A biosynthesis, particularly mutations in the close PANK3 paralog PANK2, are associated with Neurodegeneration with Brain Iron Accumulation (NBIA) or Hallervorden-Spatz syndrome, a recessive neurological disorder. Notably, known variants include PANK2 p.Ile501Thr which is at the equivalent position to PANK3 Ile301^38^. Introducing human wild-type PANK3 to PANK2 equivalent knockout (fbl^-/-^) Drosophila can partly rescue the WT phenotype^39^ suggesting some equivalency of these close paralogs. The absence of homozygous individuals despite so many heterozygous carriers makes it tempting to suggest that this PANK3 mutation could cause a similar recessive condition.

Blood group Rh(D) polypeptide (RHD) variant p.Tyr311Ser is never homozygous despite occurring in 623 and 1080 times in 1 kG and gnomAD respectively. RHD is a non-transporting homolog of other transporters (e.g. RHCG) with which it forms heterotrimers that together are involved in ammonium transport between erythrocytes and kidney/liver^40^. The crystal structure of RHCG^41^ shows that the equivalent of Tyr311 (Tyr323) lies at the protein-membrane interface, with several intramolecular hydrophobic contacts to other protein residues (Fig. 3d). In orthologs and indeed wider homologs this position is nearly always hydrophobic, is never Serine and curiously whether the position is Tyr or Cys appears to indicate whether the protein is RHD or the close paralog RHCE, respectively. Serine is disfavoured at membrane interfaces, so replacing Tyr with Ser at this position could alter the membrane position or the trimer structures^41^. At least 14 variants in RHD (magenta in Fig. 3d) have been previously associated with the “weak D antigen”^42^ of which 8 are concentrated in a region around position 311 (residues 270-339). It is plausible that two copies of this mildly or fully dysfunctional RHD subunit could lead to a disease phenotype.

Our variant impact score filtering is stringent, meaning there could well be additional promising candidates in the excluded variants. For instance, the filtered variant p.Ser244Gly in placental Alkaline Phosphatase (ALPP) is exclusively heterozygous (counts of 42 and 361 in 1 kG/gnomAD) and lies close in sequence and structure to Hypophosphatasia (HOPS) variant positions in the close paralog ALPL. These variants are associated with reduced enzyme activity^43,44^. Elsewhere, the sperm head protein Zonadhesin (ZAN) has an exclusively heterozygous variant p.Leu871Pro (43/1155 het counts and zero hom counts in 1 kG/gnomAD) that lies in an unusual repeat region in extracellular portion of this long protein. Defects in this protein might be affect to prevent haploid sperm from penetrating the egg, making it then impossible for a homozygous individual to arise.

It is possible that homozygous variants are lacking for these examples because of clear illness or disability in living persons (hence precluding inclusion in a healthy/control dataset). For certain conditions (e.g. ciliopathies only affecting certain tissues in later life), the disorder might simply not be known, which might be the case for cases such as CCDC8 where there are possibly a small number of homozygous individuals. Equally possible is that the homozygous variants are so severe that individuals are entirely unviable. This possibility is easier to argue for variants such as those in PANK3 or RHD that have more than 1000 heterozygous despite zero homozygous counts. Another possibility is that some of these variants might be beneficial when heterozygous despite being detrimental when homozygous (as with the sickle cell homozygous β-globin variant p.Glu7Val that provides malaria resistance when heterozygous^45,46^).

Evolutionary population genetics argues that deleterious variants are eventually removed from a population by purifying selection. If indeed homozygous variants are not viable or diseased, then necessarily carriers will have a lower fitness. Why are these variants still in the population? It has been argued that humans are indeed undergoing purifying selection^47–49^ and certain recessive diseases are probably examples^50–52^ of those in the process of being removed (e.g. SMA1^53,54^, IMD31B^55,56^, NSHPT^57,58^). It is difficult to argue that these candidate genes are in this category, though several of them (41/286, 14.3%) show enrichment in human sub-populations (Supplementary Table 1). For instance, NLRP12 p.Asn394Lys is twice as frequent in American, East-Asian and European populations compared with African populations, PANK3 p.Ile301Phe has a frequency of 11-14% in non-African populations i.e. 2-3 times that of African populations (5%). It is plausible that these variants were fortuitously enriched in original migratory populations.

It remains a possibility that experimental or technical artefacts are the reason why these variants are in the current databases. We managed to rule out most obvious genome features that could give rise to our observations, though there could well be oddities of epigenetics/genome modifications of which we are currently unware. We believe that requiring consistency between gnomAD and 1 kG rules out individual database biases, though this does not eliminate systematic methodological problems that might arise during variant calling and other processing steps.

There are clear experiments that could test the validity of these hypotheses. For instance, enzymes, such as PANK3, have been probed biochemically, including a number of site-directed mutants^59^. Our observations would suggest that p.Ile301Phe would alter the active site of the enzyme with effects likely observable through enzymology. It would also be interested to test cells engineered to be homozygous for these variants using CRISPR/Cas9 or similar genome tools; we would predict that many would have observable phenotypes.

Our findings, like several others^60–62^, demonstrate the power of exploiting healthy genomes to identify potentially new insights into diseases and molecular function. Our few variants above are a subset of a bigger number of variants occurring in ostensibly healthy people that drastically alter protein function. Their existence raises wider questions about gene/protein function and evolution and additional investigations will likely be highly illuminating. As databases continue to grow, more variants like those described here will be uncovered providing a potentially powerful resource to diagnose and understand human genetic diseases.

## Methods

### Data and processing

We extracted missense SNVs from variant call files (VCFs) from the 1 kG (Phase 3, 2,504 individuals) and gnomAD (V2.1.1, 125,748 exomes). We considered only those 1 kG variants where data were available in gnomAD and where the difference in Minor Allele Frequency (MAF) was ≤10%. We also converted MAF > 50% by inverting them (100-MAF). Within 1 kG we also considered other variant types (synonymous, in-frame indels, frameshifts, stop-gains, UTR, splicing, intronic or non-coding) for comparison. For easier visual comparison with missense counts in Fig. 1, the plots in Supplementary Figure 1 were created by randomly selecting up to 11k variants showing homozygous vs heterozygous counts for each variant.

### Defining exclusively nonhomozygous variants

The parental genotype in 1 kG data is unavailable. If it is assumed that homozygous variant carriers are viable and able to reproduce, the chance of homozygous offspring is approximately 0.29 after combination of all possible G0 constellations (AA x AA, Aa x Aa, AA x aa, aa x aa, Aa x AA and Aa x aa with a = WT trait and A = variant trait). When the assumption is that homozygous variant carriers are non-viable and unable to reproduce, the chance of homozygous offspring shrinks to 0.083 after the combination of all remaining G0 constellations (Aa x Aa, aa x aa, Aa x aa). The distribution of heterozygous and homozygous counts present in 1 kG generally follows this regimen (Fig. 1a). We used the Mendelian laws of inheritance-based likelihood for homozygous offspring to subject each 1 kG variant to binomial testing, defining the minimum requirement of ≥ 41 heterozygotes with 0 homozygotes for variants to be further considered.

We removed genes and their associated variants if the proteins were repeat prone (based on literature reviews; mucins, filaggrin) or more prone to mutation (olfactory receptors) or if they had any match (E ≤ 0.001 and ≥80% protein sequence identity by TBLASTN^63^ to the set of 22334 (of 204563 cDNAs) pseudogenes annotated in Ensembl^64^ version 106.

### Orthologs and alignments

We computed the orthologs for all proteins in the Uniprot proteome of Human (Proteome ID - UP000005640; retrieved April 2021) using the Orthofinder program^65^. Briefly, we used the canonical proteomes of Human and 507 other organisms from across the tree of life to compute the orthologs. In the Orthofinder program, we used the option of computing multiple sequence alignments to build gene trees and supplied an in-house species tree (will be published elsewhere) to infer the orthologs for each species pair. Next, for every protein in the Human proteome, we gathered all the orthologs across species allowing for one-to-one, one-to-many and many-to-many relationships. As mentioned above, the Orthofinder program also calculated the multiple sequence alignments for each *Orthogroup* (homologous group containing orthologs and paralogs). The alignments were calculated using the MAFFT L-INS-i method when there were <500 sequences in a group and the native MAFFT method^66^ for larger groups. To obtain the alignments for orthologs, we subset the Orthogroup alignments for each Human protein and its respective orthologs, and removed any positions that contained all gaps.

### Shuffling the 1 kG variants

A random value between 0 and 1 was chosen based on a uniform pseudo-random number generating algorithm^67^ and compared with the observed allele frequency for a given variant, with a decimal smaller/equal to the observed allele frequency determined to yield a mutated allele. For example: an allele with a variant frequency of 19 % would be considered mutated when the random number would lie between 0 and 0.19. Simulated individual genotypes would then consist of two consecutively shuffled alleles. A total of 2504 individuals (5008 alleles) were subjected to this shuffling and each of the 201k variants would undergo 100 simulation cycles. Then, the average simulated genotype would be calculated from the heterozygous and homozygous counts for each variant from each cycle.

### An integrated score for variant impact on protein function

We used alignments of orthologs (feature name *ortho*) and all homologs (*homo*) to compute HMMer profiles^68^ which provided log odds scores for each amino acid and each position. The score for any mutated position was taken as the difference between the mutated value in these profiles and the wild-type. We also used scores from the BLOSUM62 matrix for each variant (*blosum*).

We used structures for all human proteins constructed by Alphafold^5^ to define a variety of structural parameters. We first computed secondary structure (sec), main-chain dihedral (*psi/phi*) angles, and accessibility (*acc*) using DSSP^69,70^. We also computed burial (*bur*) as the accessibility of a Gly-X-Gly tripeptide minus the DSSP accessibility value. Note that burial and accessibility are thus not direct equivalents as the amino acid size affects them differently. For these commodities we then studied amino acids in representatives (fourth level of the hierarchy) of the ECOD database^71^ (v281) to first define divisions into zones: secondary structure: helical (characters H,G) strand (E,B), or coil (others); dihedral angles: a 12 × 12 grid with phi and psi (−180 – 180) in increments of 30. accessibility: low (0-15), medium (16-59), (≥60); burial: low (0-114), medium (115-164), high (≥165). We then computed log-odds scores of observed counts versus expected (based on the abundance of amino acids and the totals in each zone). For every variant we then computed the score for each commodity as log-odds mutant – log-odds wild-type) where negative values indicate a poorer fit for the mutant and vice versa. For structural parameters using Alphafold data we did not consider how the confidence scores and quality will affect wild-type and mutants equally. We also used the impact score (*mech*) from Mechismo^72^ for each variant and devised an equivalent (*mech-intra*) score using residue pair-potentials for intramolecular (in contrast to intermolecular) contacts across the ECOD dataset.

Information about approved drug targets was retrieved from the U.S. Food & Drug Administration^73^, scoring genes where medications were already approved for and when the gene was listed as disease causing in the Online Mendelian Inheritance in Man database^4^ with a (*FDA* or *OMIM*) score of 1.

For each gene we considered existing annotations on haplotype insufficiency, retrieved from ClinGen^74,75^. Genes that were associated with an autosomal recessive phenotype received a (*haplotype*) score of 1 and decreased to 0.75 when sufficient information was available or to 0.5 and 0.25 when some or only minimal information was available. Absence of information or unlikeliness for dosage sensitivity scored 0.

Lastly, we collected information about post-translation modifications, active centers and known variants from UniProt^7^ (*uniprot-function*).

We combined all of these scores (*ortho, homo, blosum, sec, acc, bur, phi/psi, mech, mech-intra, FDA, OMIM, haplotype, uniprot-function*) into a combined functional impact score using Bayesian integration^76,77^,

1 $$log_2\left( {O_{prior}} \right) + \mathop {\sum}\limits_{i = 1}^N {log} _2\left( {\frac{{\left( {D_i|P_{true}} \right.}}{{D_i|P_{false}}}} \right)$$

where $D_i|P_{true}$ and $D_i|P_{false}$ correspond to the true and false positive rates (TPR and FPR), which were obtained from ROC curves considering 26767 known disease causing variants from ClinVar as positives and a 4103 as negatives. We set $O_{prior} = 1$, arbitrarily, as we were only interested in the ranking of values and not the absolute number.

We also compared this new value to values for PMUT^9^, PolyPhen2^8^ and SIFT^10^ on the same dataset (Supplementary Figure 2b).

### Reporting Summary

Further information on research design is available in the Nature Research Reporting Summary linked to this article.

## Supplementary information

Supplementary Figures

Supplementary Dataset

Reporting Summary Checklist

## Data Availability

The 1 kG, gnomAD and additional datasets (i.e. ClinVar, UniProt and others) are publicly accessible. Remaining data generated during this study are included within the published article and its supporting information and are additionally available from the corresponding author upon request and in accordance with the Data Usage Agreement.

## Code availability

Data were processed using publicly available tools described in the Methods. Custom perl and python scripts used in this work to filter and score the resulting data can be obtained upon reasonable request from the corresponding author.

## References

[1] The 1000 Genomes Project Consortium. A global reference for human genetic variation. *Nature* **526**, 68–74 (2015). 10.1038/nature15393 PMC4750478 26432245

[2] Karczewski KJ (2020). The mutational constraint spectrum quantified from variation in 141,456 humans. Nature.

[3] Mendell JT, Dietz HC (2001). When the Message Goes Awry. Cell.

[4] McKusick-Nathans Institute of Genetic Medicine, Johns Hopkins University (Baltimore, M. Online Mendelian Inheritance in Man, OMIM®. https://omim.org/.

[5] Jumper J (2021). Highly accurate protein structure prediction with AlphaFold. Nature.

[6] Varadi, M. *et al*. AlphaFold Protein Structure Database: massively expanding the structural coverage of protein-sequence space with high-accuracy models. *Nucleic Acids Res*. 10.1093/nar/gkab1061 (2021). 10.1093/nar/gkab1061 PMC8728224 34791371

[7] UniProt: a worldwide hub of protein knowledge. *Nucleic Acids Res*. **47**, D506–D515 (2019). 10.1093/nar/gky1049 PMC6323992 30395287

[8] Adzhubei IA (2010). A method and server for predicting damaging missense mutations. Nat. Methods.

[9] López-Ferrando V, Gazzo A, de la Cruz X, Orozco M, Gelpí JL (2017). PMut: a web-based tool for the annotation of pathological variants on proteins, 2017 update. Nucleic Acids Res..

[10] Sim, N. L. et al. SIFT web server: Predicting effects of amino acid substitutions on proteins. *Nucleic Acids Res*. **40**, W452–7 (2012). 10.1093/nar/gks539 PMC3394338 22689647

[11] Huang DW, Sherman BT, Lempicki RA (2009). Systematic and integrative analysis of large gene lists using DAVID bioinformatics resources. Nat. Protoc..

[12] Boldt K (2016). An organelle-specific protein landscape identifies novel diseases and molecular mechanisms. Nat. Commun..

[13] Yan J (2014). The 3M Complex Maintains Microtubule and Genome Integrity. Mol. Cell.

[14] Hanson D, Murray PG, Black GCM, Clayton PE (2011). The Genetics of 3-M Syndrome: Unravelling a Potential New Regulatory Growth Pathway. Horm. Res. Paediatr..

[15] Mészáros B, Erdős G, Dosztányi Z (2018). IUPred2A: context-dependent prediction of protein disorder as a function of redox state and protein binding. Nucleic Acids Res..

[16] Wang P (2019). Impaired plasma membrane localization of ubiquitin ligase complex underlies 3-M syndrome development. J. Clin. Invest..

[17] Hanson D (2012). Mutations in CUL7, OBSL1 and CCDC8 in 3-M syndrome lead to disordered growth factor signalling. J. Mol. Endocrinol..

[18] Nie J (2015). Ankyrin Repeats of ANKRA2 Recognize a PxLPxL Motif on the 3M Syndrome Protein CCDC8. Structure.

[19] Hornbeck PV (2015). PhosphoSitePlus, 2014: mutations, PTMs and recalibrations. Nucleic Acids Res..

[20] Scoville DW, Kang HS, Jetten AM (2017). GLIS1-3: emerging roles in reprogramming, stem and progenitor cell differentiation and maintenance. Stem Cell Investig..

[21] Lee S-Y (2017). Glis family proteins are differentially implicated in the cellular reprogramming of human somatic cells. Oncotarget.

[22] Masetti R, Bertuccio SN, Pession A, Locatelli F (2019). CBFA2T3-GLIS2-positive acute myeloid leukaemia. A peculiar paediatric entity. Br. J. Haematol..

[23] Hara Y (2020). Patients aged less than 3 years with acute myeloid leukaemia characterize a molecularly and clinically distinct subgroup. Br. J. Haematol..

[24] Palencia-Campos A (2017). GLI1 inactivation is associated with developmental phenotypes overlapping with Ellis–van Creveld syndrome. Hum. Mol. Genet..

[25] Twigg SRF (2015). Gain-of-Function Mutations in ZIC1 Are Associated with Coronal Craniosynostosis and Learning Disability. Am. J. Hum. Genet..

[26] Roessler E (2009). The full spectrum of holoprosencephaly-associated mutations within the ZIC2 gene in humans predicts loss-of-function as the predominant disease mechanism. Hum. Mutat..

[27] Hildebrandt F, Attanasio M, Otto E (2009). Nephronophthisis: Disease Mechanisms of a Ciliopathy. J. Am. Soc. Nephrol..

[28] Halbritter J (2013). Identification of 99 novel mutations in a worldwide cohort of 1,056 patients with a nephronophthisis-related ciliopathy. Hum. Genet..

[29] Tuladhar S, Kanneganti T-D (2020). NLRP12 in innate immunity and inflammation. Mol. Asp. Med..

[30] Zhang X, Nan H, Guo J, Liu J (2021). NLRP12 reduces proliferation and inflammation of rheumatoid arthritis fibroblast-like synoviocytes by regulating the NF-κB and MAPK pathways. Eur. Cytokine Netw..

[31] Jeru I (2008). Mutations in NALP12 cause hereditary periodic fever syndromes. Proc. Natl Acad. Sci..

[32] Perez JM (2003). β1-adrenergic receptor polymorphisms confer differential function and predisposition to heart failure. Nat. Med..

[33] Riis-Vestergaard MJ (2020). Beta-1 and Not Beta-3 Adrenergic Receptors May Be the Primary Regulator of Human Brown Adipocyte Metabolism. J. Clin. Endocrinol. Metab..

[34] Inoue A (2019). Illuminating G-Protein-Coupling Selectivity of GPCRs. Cell.

[35] Yao J, Subramanian C, Rock CO, Jackowski S (2019). Human pantothenate kinase 4 is a pseudo-pantothenate kinase. Protein Sci..

[36] Zhou B (2001). A novel pantothenate kinase gene (PANK2) is defective in Hallervorden-Spatz syndrome. Nat. Genet..

[37] El-Gebali S (2019). The Pfam protein families database in 2019. Nucleic Acids Res..

[38] Hayflick SJ (2003). Genetic, Clinical, and Radiographic Delineation of Hallervorden–Spatz Syndrome. N. Engl. J. Med..

[39] Wu Z, Li C, Lv S, Zhou B (2009). Pantothenate kinase-associated neurodegeneration: insights from a Drosophila model. Hum. Mol. Genet..

[40] Van Kim C, Le, Colin Y, Cartron J-P (2006). Rh proteins: Key structural and functional components of the red cell membrane. Blood Rev..

[41] Gruswitz F (2010). Function of human Rh based on structure of RhCG at 2.1 A. Proc. Natl Acad. Sci..

[42] Wagner FF (1999). Molecular basis of weak D phenotypes. Blood.

[43] Taillandier A (1999). Characterization of eleven novel mutations (M45L, R119H, 544delG, G145V, H154Y, C184Y, D289V, 862+5A, 1172delC, R411X, E459K) in the tissue-nonspecific alkaline phosphatase (TNSALP) gene in patients with severe hypophosphatasia. Mutations in brief no. 217. Hum. Mutat..

[44] Mumm S (2002). Denaturing gradient gel electrophoresis analysis of the tissue nonspecific alkaline phosphatase isoenzyme gene in hypophosphatasia. Mol. Genet. Metab..

[45] Jha AN, Mishra H, Verma HK, Pandey I, Lakkakula BVKS (2018). Compound Heterozygosity of β-Thalassemia and the Sickle Cell Hemoglobin in Various Populations of Chhattisgarh State, India. Hemoglobin.

[46] Hedrick PW (2011). Population genetics of malaria resistance in humans. Heredity (Edinb.)..

[47] Ruiz-Pesini E, Mishmar D, Brandon M, Procaccio V, Wallace DC (2004). Effects of purifying and adaptive selection on regional variation in human mtDNA. Science.

[48] Bustamante CD (2005). Natural selection on protein-coding genes in the human genome. Nature.

[49] Zeng J (2018). Signatures of negative selection in the genetic architecture of human complex traits. Nat. Genet..

[50] Kryukov GV, Pennacchio LA, Sunyaev SR (2007). Most rare missense alleles are deleterious in humans: implications for complex disease and association studies. Am. J. Hum. Genet..

[51] Quintana-Murci L, Barreiro LB (2010). The role played by natural selection on Mendelian traits in humans. Ann. N.Y. Acad. Sci..

[52] Quintana-Murci L (2016). Understanding rare and common diseases in the context of human evolution. Genome Biol..

[53] Butchbach, M. E. R. Copy Number Variations in the Survival Motor Neuron Genes: Implications for Spinal Muscular Atrophy and Other Neurodegenerative Diseases. *Front. Mol. Biosci*. **3**, 7 (2016). 10.3389/fmolb.2016.00007 PMC4785180 27014701

[54] Wirth B (1997). De Novo Rearrangements Found in 2% of Index Patients with Spinal Muscular Atrophy: Mutational Mechanisms, Parental Origin, Mutation Rate, and Implications for Genetic Counseling. Am. J. Hum. Genet..

[55] Dupuis S (2003). Impaired response to interferon-alpha/beta and lethal viral disease in human STAT1 deficiency. Nat. Genet..

[56] Boisson-Dupuis S (2012). Inborn errors of human STAT1: allelic heterogeneity governs the diversity of immunological and infectious phenotypes. Curr. Opin. Immunol..

[57] Vahe C (2017). Diseases associated with calcium-sensing receptor. Orphanet J. Rare Dis..

[58] Herberger AL, Loretz CA (2013). Vertebrate extracellular calcium-sensing receptor evolution: selection in relation to life history and habitat. Comp. Biochem. Physiol. Part D. Genomics Proteom..

[59] Leonardi R (2010). Modulation of Pantothenate Kinase 3 Activity by Small Molecules that Interact with the Substrate/Allosteric Regulatory Domain. Chem. Biol..

[60] Abouelhoda M, Faquih T, El-Kalioby M, Alkuraya FS (2016). Revisiting the morbid genome of Mendelian disorders. Genome Biol..

[61] Möller M, Hellberg Å, Olsson ML (2018). Thorough analysis of unorthodox ABO deletions called by the 1000 Genomes project. Vox Sang..

[62] Peng T, Wang L, Li G (2017). The analysis of APOL1 genetic variation and haplotype diversity provided by 1000 Genomes project. BMC Nephrol..

[63] Altschul SF, Gish W, Miller W, Myers EW, Lipman DJ (1990). Basic local alignment search tool. J. Mol. Biol..

[64] Cunningham F (2022). Ensembl 2022. Nucleic Acids Res..

[65] Emms DM, Kelly S (2019). OrthoFinder: phylogenetic orthology inference for comparative genomics. Genome Biol..

[66] Katoh K, Standley DM (2013). MAFFT Multiple Sequence Alignment Software Version 7: Improvements in Performance and Usability. Mol. Biol. Evol..

[67] Matsumoto M, Nishimura T (1998). Mersenne twister. ACM Trans. Model. Comput. Simul..

[68] HMMER. http://hmmer.org/.

[69] Touw WG (2015). A series of PDB-related databanks for everyday needs. Nucleic Acids Res..

[70] Kabsch W, Sander C (1983). Dictionary of protein secondary structure: Pattern recognition of hydrogen-bonded and geometrical features. Biopolymers.

[71] Cheng H (2014). ECOD: An Evolutionary Classification of Protein Domains. PLoS Comput. Biol..

[72] Betts MJ (2015). Mechismo: predicting the mechanistic impact of mutations and modifications on molecular interactions. Nucleic Acids Res..

[73] U. S. Food and Drug Administration/Center for Drug Evaluation and Research. https://www.fda.gov/drugs/drug-approvals-and-databases/drugsfda-data-files.

[74] Rehm HL (2015). ClinGen — The Clinical Genome Resource. N. Engl. J. Med..

[75] Thaxton, C. et al. Utilizing ClinGen gene-disease validity and dosage sensitivity curations to inform variant classification. *Hum Mutat*. **8**, 1031–1040 (2022). 10.1002/humu.24291 PMC9035475 34694049

[76] van der Lee R (2015). Integrative Genomics-Based Discovery of Novel Regulators of the Innate Antiviral Response. PLOS Comput. Biol..

[77] Pagliarini DJ (2008). A Mitochondrial Protein Compendium Elucidates Complex I Disease Biology. Cell.

[78] Waterhouse AM, Procter JB, Martin DMA, Clamp M, Barton GJ (2009). Jalview Version 2−a multiple sequence alignment editor and analysis workbench. Bioinformatics.

[79] VMD is developed with NIH support by the Theoretical and Computational Biophysics group at the Beckman Institute, University of Illinois at Urbana-Champaign. https://www.ks.uiuc.edu/Overview/acknowledge.html.

[80] Russell RB, Barton GJ (1992). Multiple protein sequence alignment from tertiary structure comparison: Assignment of global and residue confidence levels. Proteins Struct. Funct. Genet..

