# Supplementary material for: Never-homozygous genetic variants in healthy populations are potential recessive disease candidates
Source: NPJ Genom Med. 2022 Sep 8;7:54. doi: 10.1038/s41525-022-00322-z (PMC9458638; doi:10.1038/s41525-022-00322-z)
Supplement: Supplementary file 1 — Supplementary Figures [file 41525_2022_322_MOESM1_ESM.pdf]

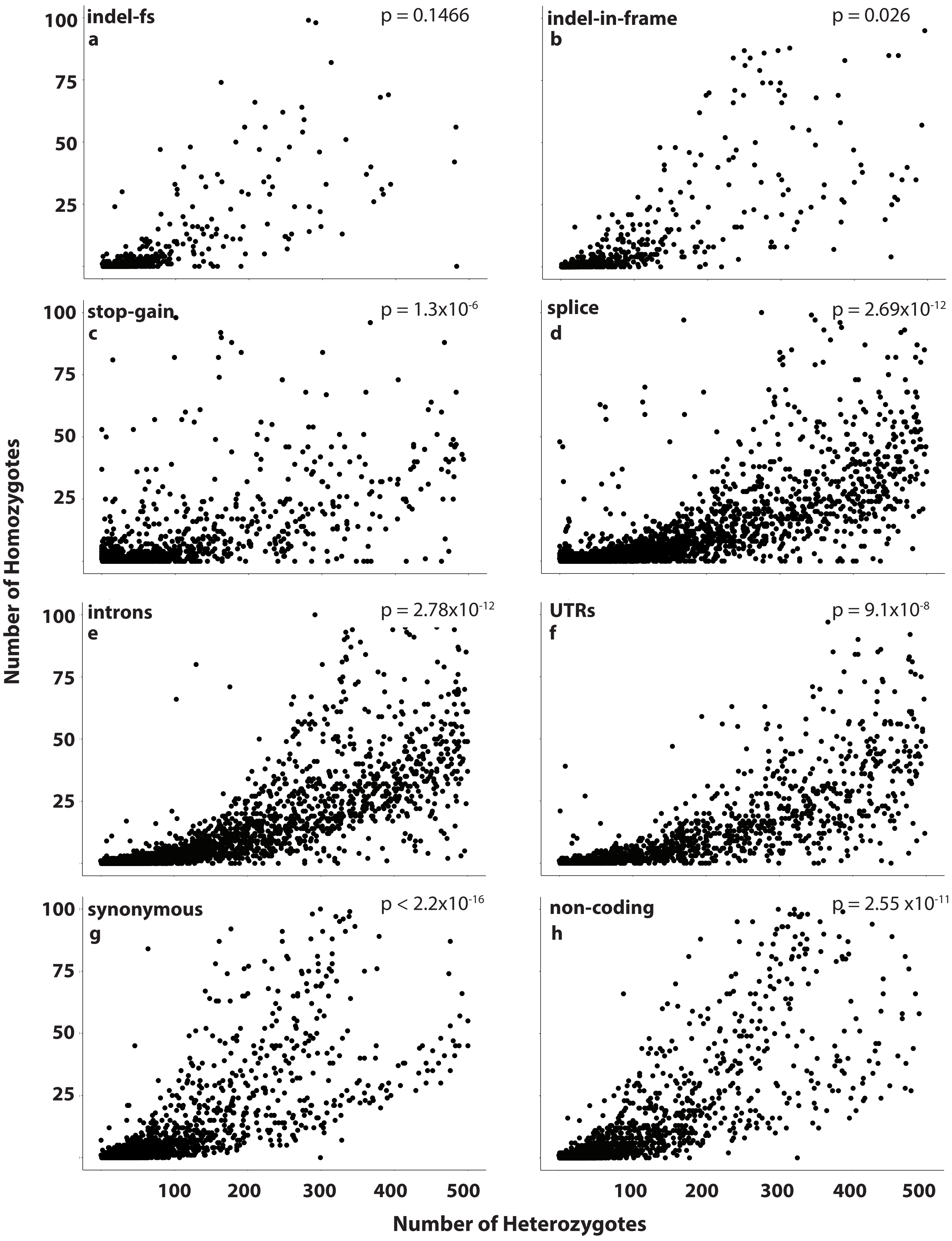

indel-fs
a
p = 0.1466
indel-in-frame
b
p = 0.026
stop-gain
c
p = 1.3x10-6
splice
d
p = 2.69x10-12
introns
e
p = 2.78x10-12
UTRs
f
p = 9.1x10-8
synonymous
g
p < 2.2x10-16
non-coding
h
p = 2.55 x10-11
Number of Homozygotes
Number of Heterozygotes
100
75
50
25
100
200
300
400
500

**Supplementary Figure 1**

**Heterozygous vs. homozygous counts for other variant types**

Plots of homozygous vs heterozygous counts for a) 1k indel-fs variants (1kG); b) 1k indel-in-frame variants; c) 11k randomly selected stop-gain variants; d) 11k randomly selected splice variants; e) 11k randomly selected intronic variants. f) 11k randomly selected UTR variants (3'-UTRs and 5'-UTRs were equally considered); g) 11k randomly selected synonymous variants; h) 11k randomly selected non-coding variants. P-values are given for each variant type comparing (Wilcoxon rank sum test) exclusively heterozygous (≥ 41) counts to those of missense variants (Figure 1a).

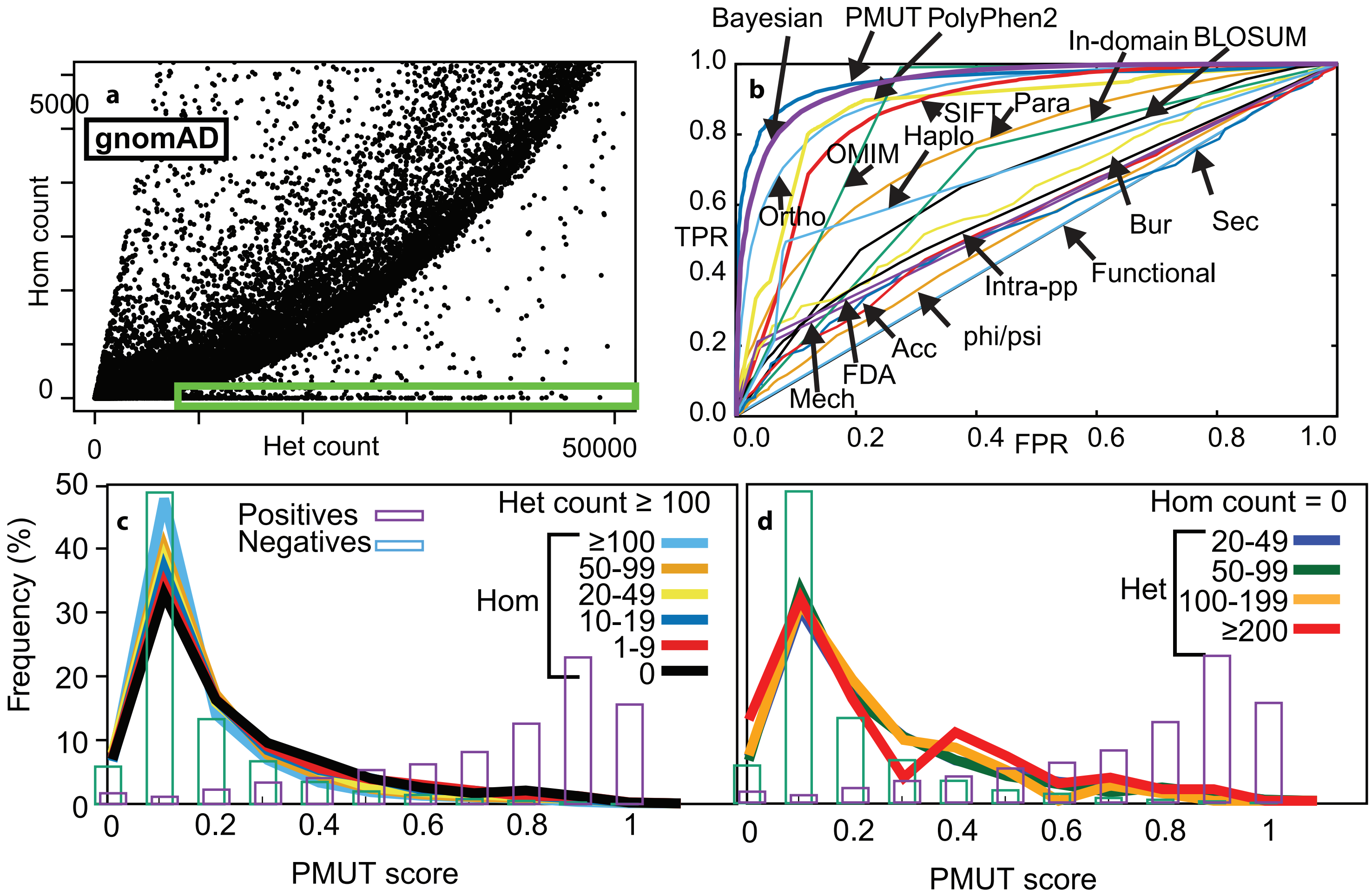


**Supplementary Figure 2**

**Exclusively heterozygous variants in gnomAD**

a) Plots of homozygous vs heterozygous counts for the gnomAD dataset. The preponderance of values on the X axis (i.e. zero homozygous counts) are indicated.
b) Receiver operator characteristic (ROC) curves (true positive rate, TPR vs false positive rate, FPR) showing how other predictors (PMUT, PolyPhen2, SIFT), the Bayesian integrated score (Bayesian) and individual components of the predictor (the others) discriminate true- (disease associated) from false-positives (SNPs excluding exclusive heterozygotes). c) Similar to Fig 1c; How the distribution of PMUT scores changes as homozygous counts decrease. d) Similar to Fig. 1d; How the distribution of PMUT scores changes for sites where homozygous counts are zero with increasing heterozygous counts.
